# 45022 Exploring Career Development Needs of Junior Investigators in Clinical Translational Science

**DOI:** 10.1017/cts.2021.562

**Published:** 2021-03-30

**Authors:** Jachael Gardner, Terry Nakazono, Jim Morrison, Kishore Athreya, Paige Hall, Pamela Davidson

**Affiliations:** David Geffen School of Medicine at UCLA

## Abstract

ABSTRACT IMPACT: By understanding Junior investigator characteristics and CTSA support services which strongly influence scientific productivity and impact, we will inform and improve research training and enhance the career development of future generations of clinical and translational science researchers. OBJECTIVES/GOALS: In the field of clinical and translational science, the career trajectory and definition of Junior Investigators (JIs) vary greatly. This study aims to investigate JI characteristics, training, and support that contribute to career development at the University of California Los Angeles (UCLA) Clinical and Translation Science Institute (CTSI). METHODS/STUDY POPULATION: Every 18 months, the UCLA CTSI administers the Longitudinal Scientific Achievement Survey, which collects information on the predictors of scientific productivity and impact. In 2018, a special supplement was added to survey JIs who received CTSA support between 2011 and 2017 (n=305), including questions on knowledge, use, and effectiveness of CTSA specific support, barriers and facilitators of research, scientific productivity, and perceived scientific impact. A literary analysis was conducted to explore previous categorizations of JIs. The JIs in our sample conducted bench to bedside, population and policy research at our four partner sites. Bivariate and logistic regression analysis were conducted to examine the significant predictors of a new grant award attributed to the CTSA support/services. RESULTS/ANTICIPATED RESULTS: The survey response rate was 82% (n=250). Respondents include core voucher co-investigators, enrollees in the Training Program in Translational Science, and K- and K-to-R workshop participants. Bivariate results showed new grant awardees significantly more likely to have the following characteristics: physician scientist with an MD and PhD (47%), pilot grant awardee (42%), core voucher awardee (49%), four or more types of CTSI support (48%), prior affiliation with an NIH institute/center other than NCATS (42%), and reported at least one impact in science, health, and/or the community (72%). Multivariate results showed that investigators with a prior core voucher award, a prior NIH affiliation, or reported one or more impacts were the strongest predictors of obtaining a new grant (each with OR>=4.0). DISCUSSION/SIGNIFICANCE OF FINDINGS: The most successful investigators consulted with NIH program officers and received feedback on their research plans and methods. Sufficient funding is crucially important to research progression. In our CTSA hub, vouchers and grants to initiate new studies or offset costs of existing research are consistent predictors of new extramural funding.

